# Integrated Approach for Biochemical and Functional Characterization of Six Clinical Variants of Glucose-6-Phosphate Dehydrogenase

**DOI:** 10.3390/ijms26178464

**Published:** 2025-08-30

**Authors:** Beatriz Hernández-Ochoa, Mónica Guadalupe Gualos-González, Jhuremy Alexandra Moreno-Hernández, Laura Morales-Luna, Montserrat Vázquez-Bautista, Luis Miguel Canseco-Ávila, Verónica Pérez de la Cruz, Roberto Arreguin-Espinosa, Elizabeth Hernández-Urzua, Sergio Enríquez-Flores, Ignacio De la Mora-De la Mora, Noemí Cárdenas-Rodríguez, Cindy Bandala, Lucia De Franceschi, Abraham Vidal-Limon, Saúl Gómez-Manzo

**Affiliations:** 1 Laboratorio de Inmunoquímica, Hospital Infantil de México Federico Gómez, Secretaría de Salud, Mexico City 06720, Mexico; beatrizhb_16@ciencias.unam.mx; 2 Laboratorio de Bioquímica Genética, Instituto Nacional de Pediatría, Secretaría de Salud, Mexico City 04530, Mexico; mggg_99@hotmail.com (M.G.G.-G.); jh.alexandra.01@gmail.com (J.A.M.-H.); lauraeloisamorales@gmail.com (L.M.-L.); montsevazquez97@gmail.com (M.V.-B.); 3 Posgrado en Ciencias Biológicas, Universidad Nacional Autónoma de México, Mexico City 04510, Mexico; 4 Programa de Posgrado en Biomedicina y Biotecnología Molecular, Escuela Nacional de Ciencias Biológicas, Instituto Politécnico Nacional, Mexico City 11340, Mexico; 5 Laboratorio de Diagnóstico y Biomedicina Molecular, Facultad de Ciencias Químicas, Campus IV, Universidad Autónoma de Chiapas, Tapachula City 30580, Mexico; cansecoavila@gmail.com; 6 Neurobiochemistry and Behavior Laboratory, National Institute of Neurology and Neurosurgery “Manuel Velasco Suárez”, Mexico City 14269, Mexico; veped@yahoo.com.mx; 7 Departamento de Química de Biomacromoléculas, Instituto de Química, Universidad Nacional Autónoma de México, Mexico City 04510, Mexico; arrespin@unam.mx; 8 Laboratorio de Toxicología Genética, Instituto Nacional de Pediatría, Secretaría de Salud, Mexico City 04530, Mexico; elyzabet91@yahoo.com.mx; 9 Laboratorio de Biomoléculas y Salud Infantil, Instituto Nacional de Pediatría, Secretaría de Salud, Mexico City 04530, Mexico; sergioenriquez@ciencias.unam.mx (S.E.-F.); ignaciodelamora@ciencias.unam.mx (I.D.l.M.-D.l.M.); 10 Laboratorio de Neurociencias, Instituto Nacional de Pediatría, Secretaría de Salud, Mexico City 04530, Mexico; noemicr2001@yahoo.com.mx; 11 Escuela Superior de Medicina, Instituto Politécnico Nacional, Mexico City 11340, Mexico; cindimiel@hotmail.com; 12 Department of Engineering for Innovative Medicine-DIMI, University of Verona, 37134 Verona, Italy; lucia.defranceschi@univr.it; 13 Red de Estudios Moleculares Avanzados, Clúster Científico y Tecnológico BioMimic^®^, Instituto de Ecología A.C. (INECOL), Carretera Antigua a Coatepec 351, El Haya, Xalapa 91073, Mexico

**Keywords:** G6PD variants, structural characterization, molecular dynamics simulations

## Abstract

Glucose-6-phosphate dehydrogenase (G6PD) deficiency is a widespread enzymopathy affecting approximately 500 million individuals that represents a significant global health issue. Among the more than 230 identified mutations in the G6PD gene, six class A variants—G6PD Utrecht (Pro409Ser), G6PD Suwalki (Pro409Arg), G6PD Merlo (Pro409Gln), G6PD Kawasaki (Gly410Ala), G6PD Shinagawa (Gly410Asp), and G6PD Riverside (Gly410Cys)—are located in the beta-loop near the NADP^+^ binding site. These mutations are of particular interest due to their association with severe hematologic phenotypes, including chronic hemolytic anemia, as well as their proposed role in the allosteric regulation of G6PD multimerization. This study presents a comprehensive biochemical and functional characterization of these clinically relevant G6PD variants. The variant enzymes were cloned, expressed, and purified for characterization. Kinetic parameters and thermal stability assays, complemented by molecular dynamics simulations (MDS), were employed to elucidate the structural impacts of the mutations. Our results demonstrate that these mutations significantly impair protein function, characterized by reduced affinity for glucose-6-phosphate (G6P) and NADP^+^, as well as altered thermal stability compared with wild-type G6PD. MDS revealed that point mutations in the βN- and βM-sheets in the NADP^+^_s_ region propagate subtle conformational changes, ultimately affecting the NADP^+^c region and the G6P binding cavity. Furthermore, secondary structure element analyses of the simulation data showed that Pro409 and Gly410 point mutations propagate several changes around residues 195–210 (G6P binding site) and 380–400 (NADP^+^_s_), explaining their effect on overall catalytic performance. These findings enhance our understanding of the molecular mechanisms underlying G6PD deficiency and its clinical implications, providing a foundation for future therapeutic strategies aimed at mitigating the effects of these variants.

## 1. Introduction

Deficiencies in enzymes that play essential roles in cellular metabolism typically lead to cellular deterioration and significant metabolic disturbances. These enzymatic defects, or enzymopathies, are inherited disorders caused by mutations in specific genes, including point mutations, insertions, deletions, and splice defects. One of the most common enzymopathies worldwide is deficiency in glucose-6-phosphate dehydrogenase (dG6PD), which affects the X-linked gene *G6PD* and leads to the generation of G6PD protein variants with different ranges of decreased enzymatic activity. This deficiency produces alterations in anaerobic metabolism, the hexose monophosphate shunt, glutathione metabolism, and the nucleotide salvage pathway in erythrocytes. Although most individuals with dG6PD are asymptomatic throughout their lives, dG6PD is associated with a wide range of biochemical phenotypes and hematological alterations [1], such as acute hemolytic anemia (AHA), neonatal jaundice (NNJ), and chronic non-spherocytic anemia (CNSA). Among these, AHA is the most common manifestation of dG6PD, triggered by drugs, ingestion of fava beans, or infections [2,3,4,5,6].

To date, more than 231 variants have been described, approximately 85% of which involve exonic mutations, causing amino acid changes and altering enzyme properties [7,8,9,10]. Notably, up to 50 mutations have been identified in exon 10 of the *G6PD* gene that involve amino acid residues located at the dimer interface, thereby exerting a negative effect on protein stability and dimerization [10]. A minority of these mutations are associated with severe hematologic phenotypes [9,10].

An evolutionary analysis based on a multiple alignment of amino acid (aa) sequences from 52 G6PD orthologs across 42 organisms revealed a correlation between aa replacements causing G6PD deficiency in humans and G6PD sequence conservation: two-thirds of such replacements affect highly and moderately conserved aas, and relatively few affect fully conserved aas and non-conserved aas, where they could be lethal, or presumably simply would not cause G6PD deficiency [11]. Furthermore, mutations in the G6PD protein are spread throughout the entire sequence. Only a discrete cluster arises in the range of aa 380–410, corresponding to the subunit interface in the enzymatically active G6PD dimer [12]. Given the large number of mutations identified in the *G6PD* gene, numerous attempts have been made to understand the link between genotype and phenotype, to help explain the impact of different mutations on G6PD enzymatic activity. Therefore, the biochemical and kinetic characterization of the variants is crucial.

**Figure 1:**
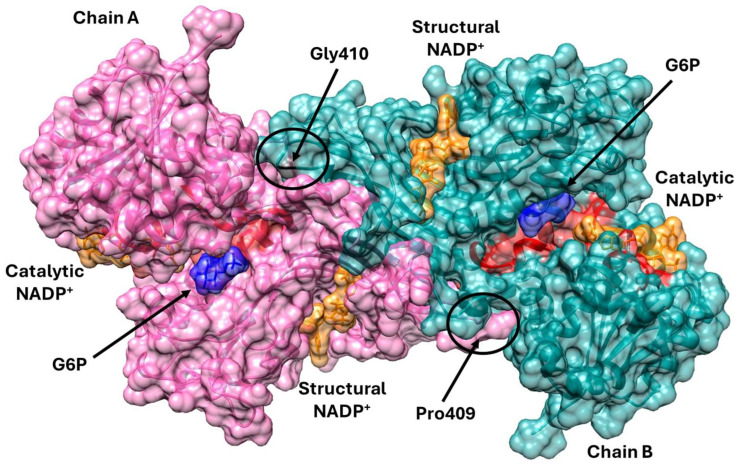
Tridimensional structure of dimeric wild-type (WT) G6PD, indicating the mutation sites. The folded α-helices and β-strands that compose the structure are visible. The two monomers, labeled Chain A and Chain B, are shown in pink and light blue, respectively. The G6P and catalytic NADP^+^ substrates are depicted in blue and orange, respectively and the catalytic site is shown in red color. The region containing Pro409 and Gly410, where the mutations occur, is highlighted. The figure was created with PyMOL Molecular Graphic System program, version 2.5.0.

The aim of this study was to analyze, by biochemical and computational methods, six G6PD variants associated with severe hemolytic phenotypes (class A). Three of these variants affect Pro409—Suwalki (Pro409Arg), Merlo (Pro409Gln), and Utrecht (Pro409Ser) [13,14,15]—and the other three affect Gly410—Kawasaki (Gly410Ala), Riverside (Gly410Cys), and Shinagawa (Gly410Asp) [16,17,18,19] (Figure 1). The clinical relevance of our findings is supported by the severity of the hematologic phenotypes that these mutations are associated with. Thus, our data go beyond the current state of knowledge by linking, for the first time, structural alterations to enzymatic dysfunction in six distinct G6PD variants that promote chronic hemolysis.

## 2. Results and Discussion

### 2.1. In Silico Analysis

#### 2.1.1. Phylogenetic Examination of the Conservation of G6PD Protein Residues

**Figure 2:**
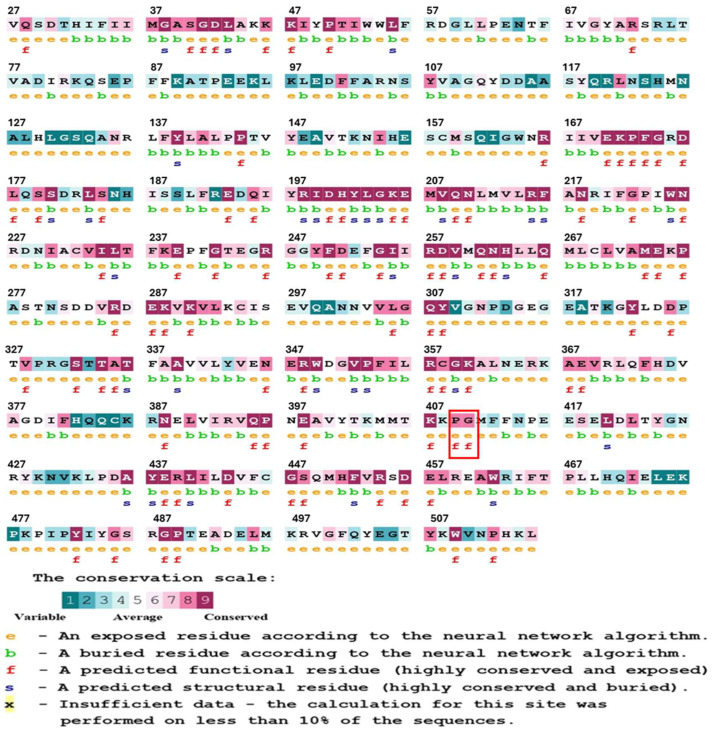
Analysis of G6PD phylogenetic conservation, generated using the ConSurf tool. The proline and glycine residues inside the red box are amino acids 409 and 410, respectively.

Evolutionary information is a useful tool in the study of protein structure and function, as it helps to identify residues that play critical roles. Amino acids that are necessary to maintain the structure of the protein, or that are involved in important functions, such as ligand binding and catalysis, tend to be evolutionarily conserved, meaning that, over time, their positions tend to change more slowly over time compared with other, less critical residues [20,21]. To assess the degree of phylogenetic conservation of residues in the G6PD protein, we conducted a conservation analysis using the ConSurf online tool [22,23] (Figure 2). The tool performs a multiple sequence alignment and assigns a conservation score to each residue. The analysis revealed that Pro409 and Gly410 are highly conserved residues, with a score of 7. Based on this conservation data, mutations of Pro409 and Gly410, which are highly conserved residues, would be detrimental to protein function.

#### 2.1.2. Analysis of the Impact of the Determined Variants on G6PD Protein Structure

To analyze the structural and functional effects of the point mutations encoding the Utrecht, Merlo, Suwalki, Kawasaki, Shinagawa, and Riverside variants of the G6PD protein, an analysis was performed using the HOPE online tool [24]. Pro409 and Gly410 are located within an important domain for the activity of the protein and are in contact with another domain. Within this pocket, there are amino acids involved in the correct positioning of the G6P substrate. It is possible that this interaction is important for the correct function of the protein. The interaction between these domains could be disturbed by the mutations, which might affect signal transduction between the domains and the correct function of the protein. G6PD variants located at Pro409 and Gly410 (two residues positioned within the β-loop near the structural NADP^+^ (NADP^+^_s_) site), are located on the surface of the protein, and mutating them could interfere with interactions involving other protein regions or molecules. Glycine, due to its flexibility, can adopt unusual torsion angles; its substitution with a different residue may force the local backbone into an unfavorable conformation, thereby disturbing the local structure.

**Figure 3:**
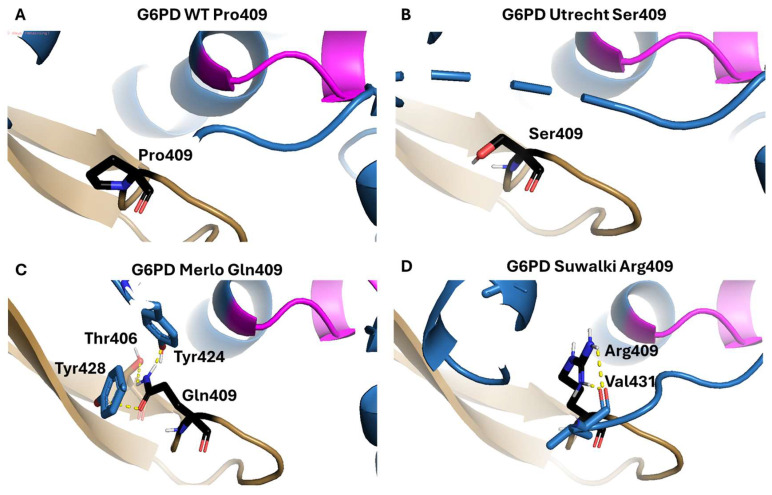
Comparison between the wild-type G6PD enzyme and the Utrecht, Merlo, and Suwalki variants. (**A**) WT-G6PD. (**B**) In silico mutation to generate the Utrecht variant. (**C**) In silico mutation to generate the Merlo variant. (**D**) In silico mutation to generate the Suwalki variant. The in silico mutations were generated using Chimera 1.16, and the substituting amino acids are presented in black. Monomers A and B are presented in blue and brown, respectively, and the pink amino acids in monomer A are those that participate in correct positioning of the G6P substrate, the hydrogen bonds are represented as yellow dotted lines.

In addition, we performed in silico mutagenesis to identify the impact of these substitutions on the protein structure. In WT-G6PD, the residues Pro409 and Gly410 are located in a β-turn structure within a solvent-exposed loop (Figure 3). The amino acid residues that most commonly favor the β-turn formation are Gly, Pro, Asn, and Asp. β-turns are secondary structures that play an important role in proteins by changing the overall direction of polypeptide chains, thereby contributing to their compact folded structures [25,26]. Furthermore, it has been reported that the Pro-Gly amino acid sequence specifically favors the formation of β-turn and β-bend structures in proteins [27,28]. Furthermore, the proline side chain, which consists of a pyrrolidone ring, confers specific structural and dynamic properties that determine several of its functions, including local disruption of α-helical segments, which are essential for folded protein structures. Furthermore, proline is also involved in the formation of surface “turns,” which direct the folding of water-soluble proteins [29]. Thus, mutation of a proline residue could cause a loss of secondary structure in the protein.

The in silico predictions showed that Pro409 does interact with neighboring amino acids. The Utrecht variant replaces Pro409 with the amino acid Ser, which has a more flexible side chain than Pro and introduces a negative charge, which could alter the stability and activity of the protein. On the other hand, when the Pro was changed to the amino acid Gln, the formation of three H bonds was observed between the Gln side chain and the amino acids Thr406, Tyr424, and Tyr428. It is important to mention that the G6PD active dimer interface is stabilized by four salt bridges between Glu206 and Lys407 and Glu419 and Arg427. Pro409 is located near these amino acids [30]; thus, replacing it with a glutamine (a polar, positively charged amino acid) likely affects these salt bridges, impairing dimer formation and, consequently, enzymatic activity. Finally, substitution of Pro with Arg, whose side chain has a guanidino group, results in two hydrogen bonds with the Val431peptide bond.

**Figure 4:**
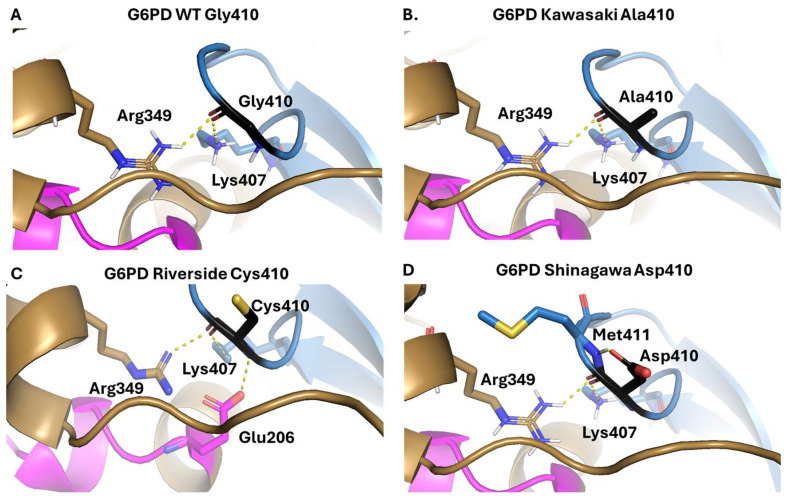
Comparison between the wild-type G6PD enzyme and the Kawasaki, Riverside, and Shinagawa variants. (**A**) WT-G6PD. (**B**) In silico mutation to generate the Kawasaki variant. (**C**) In silico mutation to generate the Riverside variant. (**D**) In silico mutation to generate the Shinagawa variant. The in silico mutations were generated using Chimera 1.16, and the substituting amino acids are presented in black. Monomers A and B are presented in blue and brown, respectively, and the pink amino acids in monomer A are those that participate in correct positioning of the G6P substrate, the hydrogen bonds are represented as yellow dotted lines.

The Gly410 residue (located in the B chain of the G6PD dimer) is predicted to participate in two interactions between the peptide bond and residues Arg349 and Lys407 of the A chain (Figure 4A). When Gly410 is substituted with Ala in the G6PD Kawasaki variant, these two hydrogen bonds remain the same as in WT G6PD. However, Ala is a larger and more rigid amino acid than Gly, which is likely to make the area more rigid, potentially leading to structural destabilization and loss of catalytic activity (Figure 4B). Meanwhile, substitution of Gly410 with a cysteine residue, which corresponds to the G6PD Riverside variant, is predicted to generate a new hydrogen bond between the nitrogen atom of the peptide bond and Glu206 (Figure 4C). This might destabilize the 3D structure of the protein, increasing its thermolability and decreasing its catalytic activity. Finally, when Gly410 is changed to Asp, as seen in the G6PD Shinagawa variant, the two hydrogen bonds remain the same as in WT G6PD (Figure 4D). Of note, Asp is a larger and more rigid amino acid than Gly and introduces a negative charge in the mutation zone, which might negatively impact the stability and catalytic ability of the protein. Mutation of Gly410 could also affect the salt bridges that stabilize the G6PD dimer (Glu206 to Lys407 and Glu419 to Arg427), which would lead to loss of the active dimer [30].

American College of Medical Genetics and Genomics and the Association for Molecular Pathology (ACMG/AMP) [31] recommendations describe a process for classifying variants identified in genes that cause Mendelian disorders into five categories. The classification is based on criteria using typical types of variant evidence (e.g., population data, computational data, functional data, segregation data). According to the ACMG/AMP, the G6PD variants Suwalki (Pro409Arg), Merlo (Pro409Gln), Utrecht (Pro409Ser), Kawasaki (Gly410Ala), Riverside (Gly410Cys), and Shinagawa (Gly410Asp) were classified as pathogenic alleles (evidence categories PS3, PM1, PM2, PM5, PP2, PP3, and PP4) (https://www.medschool.umaryland.edu/Genetic_Variant_Interpretation_Tool1.html/, accessed on 11 July 2025). This is consistent with clinical manifestations reported in patients with these variants, such as intensive jaundice in the neonatal period, chronic non-spherocytic hemolytic anemia (CNSHA), and low or no G6PD activity in red blood cells. To understand how mutations impact protein stability, we used DDMut, a fast and accurate Siamese network that predicts changes in Gibbs free energy (ΔΔG) for single and multiple point mutations. DDMut incorporates deep learning models that combine graph-based 3D environmental representations with convolutional layers and transformer encoders to understand mutations. This approach enhances the capture of both short-range and long-range atomic interactions, offering an alternative for predicting mutation effects on protein stability [32]. We aimed to predict the impact of single mutations on Pro409 and Gly410 to integrate a more robust explanation of G6PD structure and function (Figure S1).

### 2.2. Plasmid Construction and Expression and Purification of Recombinant Human G6PD

To construct the six clinically relevant G6PD variants (Suwalki (Pro409Arg), Merlo (Pro409Gln), Utrecht (Pro409Ser), Kawasaki (Gly410Ala), Riverside (Gly410Cys), and Shinagawa (Gly410Asp)), we performed site-directed mutagenesis, introducing the mutations into the pET-3a plasmid containing the wild-type *G6PD* gene sequence, confirmed by sequencing. Then, the pET-3a plasmids containing each mutated variant were transformed into *E. coli* BL21(DE3)Δzwf::kan^r^ expression cells. The six variants were purified by anion exchange chromatography (Q-Sepharose 4B column) followed by affinity chromatography (2′,5′-ADP Sepharose 4B affinity column). The purity of all recombinant G6PD proteins was greater than 90%. The total protein concentrations obtained for each variant were as follows: Suwalki (0.2 mg/mL), Merlo (0.64 mg/mL), Utrecht (0.1 mg/mL), Kawasaki (4.4 mg/mL), Shinagawa (1.28 mg/mL), and Riverside (0.64 mg/mL). It is essential to mention that the yields of G6PD variants with mutations affecting Pro409 were lower than those of variants with mutations affecting Gly410. However, all of the G6PD variants yielded lower total concentrations of purified protein compared with the native G6PD enzyme (Table S1). These results suggest that these mutations, which are located near the dimer interface, negatively affect G6PD expression, probably by causing the protein to not be folded correctly during its synthesis, affecting the stability of these G6PDs variants.

### 2.3. Functional Characterization

To determine the effects of the mutations on the catalytic activity of the native (WT-G6PD) enzyme, functional evaluation of the active site was carried out through steady-state kinetic experiments and thermal inactivation assays for the six clinical variants: G6PD Suwalki (Pro409Arg), Merlo (Pro409Gln), Utrecht (Pro409Ser), Kawasaki (Gly410Ala), Riverside (Gly410Cys), and Shinagawa (Gly410Asp).

#### 2.3.1. Measurement of Steady-State Kinetic Parameters

To evaluate the effects of the mutations on the catalytic activity of the native (WT-G6PD) enzyme, the steady-state kinetic parameters K_m_, *k*_cat_, and *V*_max_ were obtained for the six clinical G6PD variants. Regarding the three clinical variants affecting Pro409 (G6PD Suwalki (Pro409Arg), Merlo (Pro409Gln), and Utrecht (Pro409Ser)), the enzyme activity compared with that of G6PD was <1%, and the total protein concentrations obtained for each variant were Suwalki (0.2 mg/mL), Merlo (0.64 mg/mL), and Utrecht (0.1 mg/mL). No kinetic data could be obtained for any of these three variants. These results are in concordance with previous observations of patients with these variants, who show severe G6PD deficiency, chronic non-spherocytic hemolytic anemia (CNSHA), and <5% residual enzyme activity. These results are also consistent with previous reports on natural Class A variants (previously Class I variants), including G6PD Bangkok and G6PD Canton + Bangkok noi [33], Guadalajara, G6PD Mount Sinai, and G6PD No name [34], in which the residual G6PD activity was <1%.

**Figure 5:**
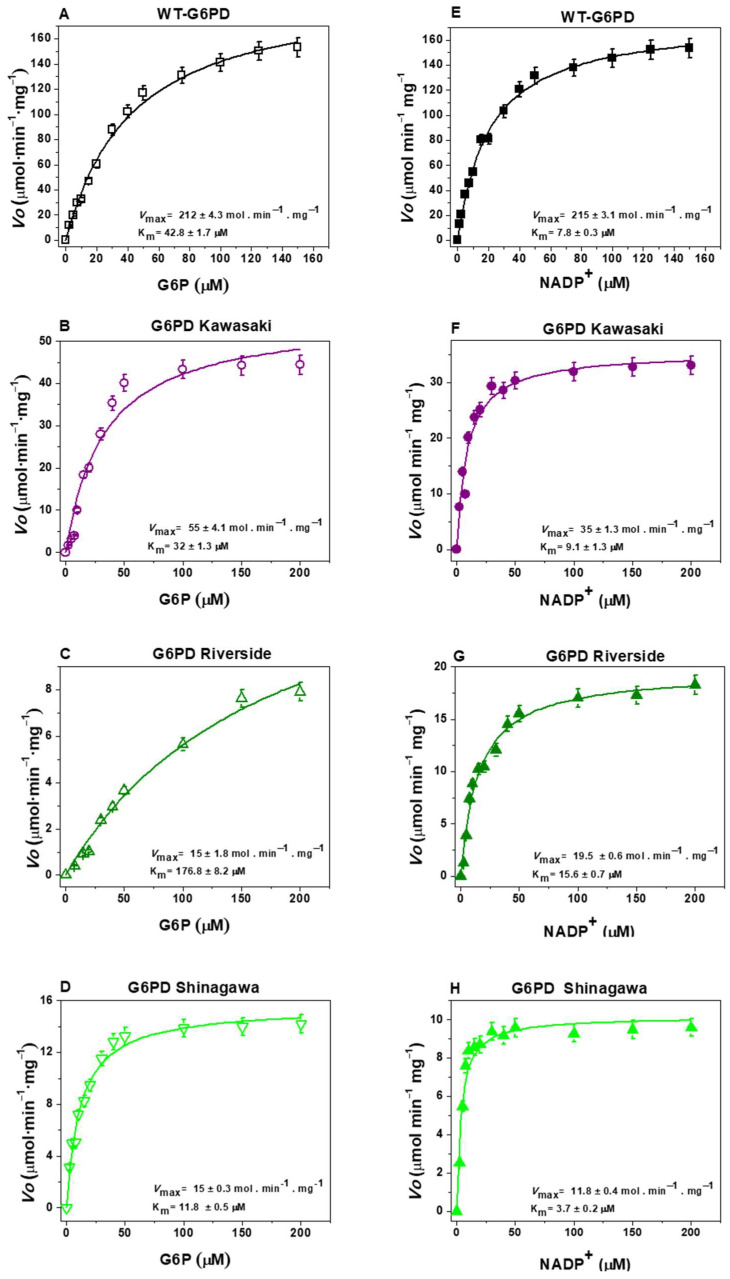
Representative kinetic plots of WT-G6PD (**A**,**E**) and three clinical G6PD mutants: (**B**,**F**) G6PD Kawasaki (Gly410Ala), (**C**,**G**) G6PD Riverside (Gly410Cys), and (**D**,**H**) G6PD Shinagawa (Gly410Asp). Initial velocity (*V*o) data were obtained by varying the concentration of the substrate indicated on the abscissa, with the second substrate fixed at a saturating concentration. The data were fitted to the Michaelis–Menten equation by non-linear regression calculations. The data represent the mean ± SD from three independent experiments.

Figure 5 shows the initial velocity values for the G6P and NADP^+^ substrates obtained for the WT-G6PD enzyme and the variants with mutations affecting Gly410: Kawasaki (Gly410Ala), Riverside (Gly410Cys), and Shinagawa (Gly410Asp). For WT-G6PD, values of K_m G6P_ = 38.4 μM and K_m NADP+_ = 6.1 μM were obtained for the G6P and NADP^+^ substrates, respectively (Figure 5A,E) (Table 1). Regarding the clinical variant G6PD Kawasaki (Gly410Ala), a value of K_m G6P_ = 32.4 μM was obtained for the G6P substrate, and a value of K_m NADP+_ = 9.1 μM was obtained for the NADP^+^ substrate (Figure 5B,F) (Table 1). For the clinical Riverside (Gly410Cys) variant, a value of K_m G6P_ = 176.8 μM was obtained for the G6P substrate, and a value of K_m NADP+_ = 15.6 μM was obtained for the NADP^+^ substrate (Figure 5C,G) (Table 1). Finally, for the clinical Shinagawa (Gly410Asp) variant, a value of K_m G6P_ = 11.8 μM was obtained for the G6P substrate, while for the NADP^+^ substrate, a value of K_m NADP+_ = 3.7 μM was obtained (Figure 5D,H) (Table 1).

To determine whether there are statistically significant differences in the kinetic parameters of the WT-G6PD enzyme and the three G6PD variants affecting position 410, we performed nonparametric tests (one-way ANOVA). The results indicated that there were significant differences in K_m_ values (for both physiological substrates) between the Riverside and Shinagawa variants and the native enzyme (*p*-value < 0.05). However, the Kawasaki variant showed a significant difference in K_m NADP_^+^ (*p*-value = 0.0007) but did not show a significant difference in K_m G6P_ (*p*-value = 0.09) compared with the WT-G6PD enzyme. These results reveal that, although the mutations are located in the same position within the three-dimensional (3D) structure of the enzyme (at codon 410), they result in different affinities for the two physiological substrates, as well as different catalysis values compared with the WT-G6PD enzyme. For the Kawasaki variant (Gly410Ala), the affinities for both physiological substrates were similar to those of the native enzyme (WT-G6PD). The Shinagawa variant (Gly410Asp) exhibited greater affinity for both physiological substrates than WT-G6PD. However, the Riverside variant (Gly410Cys) showed less affinity for the two physiological substrates. This same effect was observed regarding catalysis (*k*_cat_), where the Kawasaki, Riverside, and Shinagawa variants exhibited less catalytic activity (34.3, 18.6, and 18.7 s^−1^, respectively) than WT-G6PD (211.3 s^−1^), indicating losses of catalytic activity of 85%, 93%, and 91% for the clinical G6PD variants Kawasaki, Shinagawa, and Riverside, respectively.

Taken together, our results indicate that these G6PD variants are not catalytically active, which is consistent with the chronic hemolytic anemia observed in patients carrying these mutations. Indeed, a cut-off of <20% G6PD activity for the identification of Class A G6PD variants has been established, highlighting the key role of G6PD in red blood cell homeostasis during physiologic oxidation throughout the lifespan of erythrocytes in the peripheral circulation [35]. The observed differences in substrate affinity and catalysis can be attributed to the physicochemical properties of the substituting amino acids. For example, in the G6PD Kawasaki (Gly410Ala) variant, glycine (an aliphatic amino acid) was replaced with alanine (also aliphatic), introducing a CH_3_ side chain into the 3D structure of the protein, which caused no significant alterations in the affinities for the two substrates compared with the WT-G6PD enzyme. In the natural G6PD Shinagawa (Gly410Asp) variant, glycine (an aliphatic amino acid) was replaced with aspartic acid (an acidic amino acid), introducing a negative charge at this position, resulting in reduced catalytic activity, despite the increased substrate affinity. Finally, in the natural G6PD Riverside (Gly410Cys) variant, glycine (an aliphatic amino acid) was replaced with cysteine (a sulfur-containing amino acid), introducing a sulfur-containing side chain, potentially disturbing the local structural conformation and impairing substrate binding and enzymatic activity.

#### 2.3.2. Thermal Inactivation Assays

**Figure 6:**
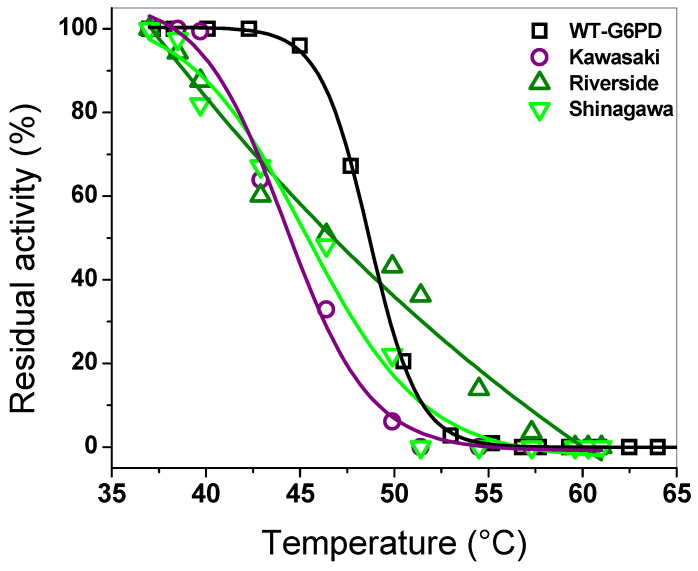
Thermal inactivation assays for three single mutants (Kawasaki, Riverside, and Shinagawa) and WT-G6PD. All the proteins were adjusted to 0.2 mg/mL and incubated at different temperatures. Residual activity was plotted as percentage of the same sample incubated at 37 °C. The assays were performed in triplicate; standard errors were lower than 5%.

An assay that is widely used to test the effect of mutations on the WT-G6PD enzyme is thermal inactivation analysis, which evaluates the thermal stability of the active site of each recombinant G6PD variant [1,9,36,37,38,39,40,41]. Here, the assays were carried out at a concentration of 10 µM of NADP^+^. As shown in Figure 6, as the temperature increased, the recombinant enzymes lost their initial activity. We determined the T_50_ values (the temperature at which the protein loses 50% of its initial activity) for each variant. The T_50_ values calculated for the Kawasaki, Shinagawa, and Riverside variants were 44, 44.5, and 46.8 °C, respectively (Figure 6), while WT-G6PD exhibited a T_50_ of 47.5 °C. These results indicate that the three clinical mutants analyzed in this experiment lost 50% of their initial activity at a lower temperature than WT-G6PD, indicating that there is a reduction in stability when the mutations are present; this is related to the clinical manifestation of chronic non-spherocytic hemolytic anemia. It is important to mention that although the mutations affect the same codon (Gly410), their effect on thermal stability differs, highlighting the influence of the specific amino acid substitutions.

### 2.4. Spectroscopic Characterization

To further explore the structural basis for the decreased enzymatic activity and thermal stability, we performed spectroscopic analyses, including circular dichroism (CD) and intrinsic and extrinsic fluorescence assays, to evaluate changes in the secondary and tertiary structure of the mutants compared with WT-G6PD.

#### 2.4.1. Circular Dichroism (CD) Analysis

**Figure 7:**
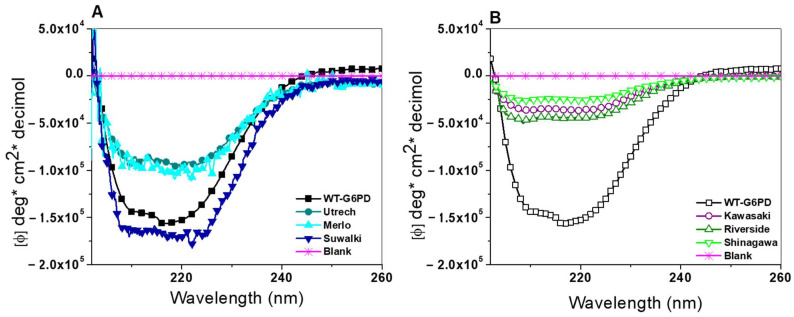
Spectroscopy characterization of the six clinical mutants and WT-G6PD. Structural characterization was performed in the far-UV region (200–260 nm) for Pro409 (**A**) and Gly410 (**B**). The experiments were performed in triplicate, and these spectra are representative of the triplicate experiments.

To assess alterations in secondary structure, we carried out CD analysis in the far-UV region (190–260 nm). As shown in Figure 7, all six G6PD variants displayed two characteristic negative peaks at 208 and 222 nm, corresponding to α-helix and β-sheet structures typical of G6PD [1,9,36,37,38,39]. Furthermore, according to the CD spectra results, a significant change was observed for all the analyzed variants compared with WT-G6PD. However, it is interesting to note that the variants located in Pro409 had a greater intensity compared with variants located in the position Gly410 (Figure 7A). The Suwalki (Pro409Arg) variant showed greater intensity than WT-G6PD (Figure 7A). This phenomenon was also observed in G6PD Asahi, G6PD Acrokorinthos, G6PD A^−(680)^, and G6PD Guadalajara, where significant changes were reported [34]. As shown in Figure 7B, the spectra of the three clinical Class A variants (formerly Class I)—G6PD Kawasaki (Gly410Ala), Shinagawa (Gly410Asp), and Riverside (Gly410Cys)—showed lower minimum absorption peaks at 208 and 222 nm compared with the signal intensity obtained for WT-G6PD, indicating a modification in the chirality of the chromophores after mutation. This confirms that the mutations affected the secondary structure of the WT-G6PD enzyme, which may explain the diminished catalytic activity observed in each of the G6PD mutants. These results agree with what was previously reported for the Class A (formerly Class 1) G6PD variants Guadalajara, Durham, Zacatecas, Yucatan, Bangkok (I), Bangkok noi (I), and Canton + Bangkok noi, which also showed very notable changes compared with WT-G6PD [33,34,39].

#### 2.4.2. Intrinsic and Extrinsic Fluorescence Assays

**Figure 8:**
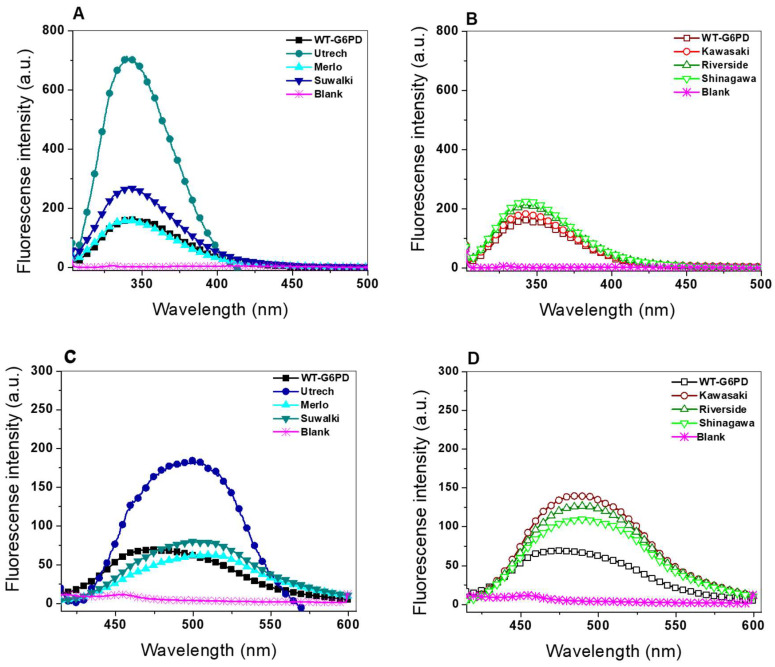
Fluorescence emission spectra of WT-G6PD and six clinical mutants. Intrinsic fluorescence spectra of WT-G6PD and Pro409 (**A**) and Gly410 (**B**) clinical G6PD variants. 8-anilinonaphthalene-1-sulphonate (ANS) assays of WT-G6PD and Pro409 (**C**) and Gly410 (**D**) clinical G6PD variants. The experimental conditions for all experiments are described in the Materials and Methods.

**Figure 9:**
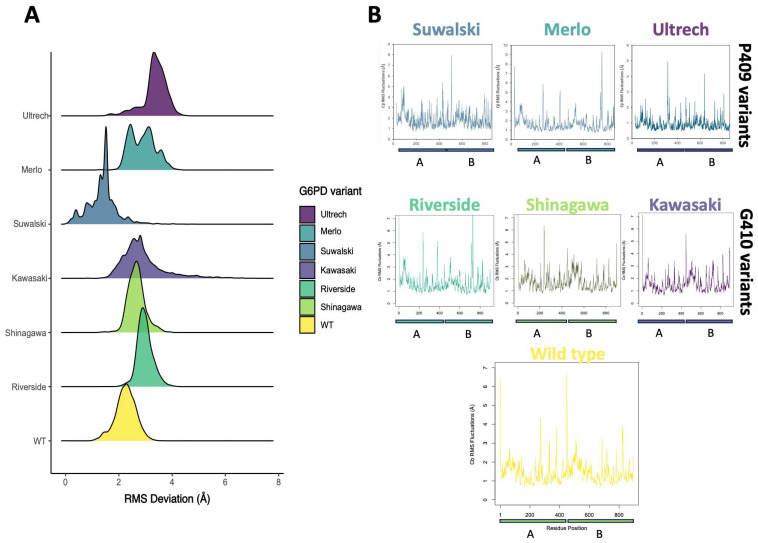
Structural characterization of Pro409 and Gly410 β-loop G6PD variants. (**A**) Cumulative 1.5 µs RMSD of G6PD dimeric Pro409 and Gly410 variant systems. RMSD is shown as a histogram distribution. (**B**) RMSF fluctuations of Pro409 and Gly410 variants. Each monomer is displayed separately to clearly assess the differences.

The intrinsic fluorescence of proteins is attributed to π to π* transitions in the indole ring of the tryptophan side chain; however, the emission intensity is susceptible to quenching by water molecules [42,43]. Thus, the emission maximum and emission intensity are highly dependent on the immediate environment surrounding the tryptophan side chain. Changes in the emission spectrum are observed in response to protein conformational transitions, ligand binding, or denaturation; likewise, the introduction of a new residue because of a mutation can affect the local environment surrounding tryptophan residues. To determine whether these structural alterations extended to the tertiary level, intrinsic and extrinsic fluorescence spectroscopy was conducted, focusing on the seven tryptophan residues per G6PD monomer. As shown in Figure 8, all variants exhibited alterations in intrinsic fluorescence compared with WT-G6PD. Among the variants affecting Pro409, G6PD Utrecht showed the highest increase in fluorescence intensity (811 a.u.), followed by G6PD Suwalki (272 a.u.), while the Merlo variant showed fluorescence intensity similar to WT-G6PD (159 a.u.) (Figure 9A). The increase in the fluorescence intensity of tryptophan residues in the G6PD Utrecht variant is similar to that previously reported for the G6PD Class A variants Zacatecas, Durham, and Veracruz, for which a two-fold was detected compared with WT-G6PD [9,36,38]. The intrinsic fluorescence results obtained for the Utrecht and Suwalki variants indicate a relative increase compared with WT-G6PD, suggesting repositioning of the tryptophan residues in a less polar environment, which suggests structural modifications in the variants.

Regarding the fluorescence intensity of the tryptophan residues for the variants with mutations affecting Gly410, the fluorescence emission spectra for all the mutants were simitar to WT-G6PD (159 a.u.) (Figure 8B). Of note, the degree of mutant fluorescence was Shinagawa (Gly → Asp) (215 a.u.) > Riverside (Gly → Cys) (197 a.u.) > Kawasaki (Gly → Cys) (165 a.u.) (Figure 8A).

These increases in the intrinsic fluorescence of tryptophan residues in all the clinical variants analyzed in this study suggested slight conformational changes in the native 3D structure of these proteins that have a negative effect on the catalytic activity of the six variants. Importantly, a correlation was observed between increased intrinsic fluorescence and reduced catalytic efficiency. in the variants with mutations affecting Pro409 position were those that presented enzyme activity < 1%, and the variants with mutations affecting the Gly410 position also exhibited a direct relationship between the increase in intrinsic fluorescence and the loss of activity: the Shinagawa mutant was the one that presented the lowest catalytic activity and lost affinity (K_m_) for its two physiological substrates, followed by the Riverside and Kawasaki mutants.

Additionally, we monitored the extrinsic fluorescence emission spectra using ANS, a probe with high affinity for hydrophobic protein surfaces. As shown in Figure 8C,D, the extrinsic fluorescence intensities of all six clinical variants were elevated compared with the WT-G6PD enzyme. This increase in ANS fluorescence suggests conformational changes in the 3D structure that expose hydrophobic regions not accessible in the native protein. The native WT-G6PD enzyme has a minimal fluorescence emission spectrum of 60 arbitrary units (a.u.) at 482 nm (Figure 8C), while the G6PD variants showed fluorescence emission spectra of 79, 182, and 62 a.u. for the G6PD variants Suwalki, Utrecht, and Merlo, respectively (Figure 8C). In addition, it is important to note that we observed a displacement in the maximal fluorescence intensity (an ANS of 10 nm was observed in the G6PD Suwalki variant). This same blue shift at a shorter wavelength was also observed in the G6PD Durham, Veracruz, Santa Maria, A+, Mexico DF, Bangkok (I), Canton + Bangkok noi (I), Songklanagarind (II), Union (II), and Canton (II) variants [33,36,38].

The Gly410 variants Shinagawa, Riverside, and Kawasaki showed fluorescence emissions of 100, 120, and 140 a.u., respectively. These results align with the previously reported in Class A (formerly Class I) variants, which also showed an increase in fluorescence intensity compared with the native enzyme and loss of activity and stability [1,9,33,34,36,37,38,39]. These findings confirm that the mutations present in Class A G6PD variants significantly affect protein stability, resulting in lower purification yields, reduced catalytic efficiency, and structural alterations at both the secondary and tertiary levels, ultimately contributing to the clinical manifestations seen in patients with G6PD deficiency.

### 2.5. Conformational Dynamics of G6PD β-Sheet Variants

To corroborate the biochemical observations, we performed conformational dynamics analyses of the G6PD β-sheet variants using molecular dynamics simulations (MDSs). G6PD is a dynamic enzyme whose activity is intricately regulated by a structural NADP^+^ (NADP^+^_s_) molecule, which binds ~25 Å away from the catalytic NADP^+^ (NADP^+^c) site. Previous reports have shown how mutations alter the equilibrium between different conformations, highlighting the importance of understanding their role in disease and drug resistance [44,45,46]. To address the effect of point mutations on the NADP+s region, we performed molecular dynamics simulations (MDSs) of seven dimeric G6PD systems: wild-type (WT), Suwalki (Pro409Arg), Merlo (Pro409Gln), Utrecht (Pro409Ser), Kawasaki (Gly410Ala), Riverside (Gly410Cys), and Shinagawa (Gly410Asp). All of these mutations are located in the β-loop region of the structural NADP^+^ (NADP^+^_s_). Throughout the cumulative 1.5 µs length of the MDS, the G6PD Gly410 variants exhibited similar flexibility/atomic deviations compared with WT-G6PD. For the variants with mutations affecting Pro409, two of them, specifically Merlo and Utrecht, showed increased alpha carbon RMS deviations (~1.5 and 2 Å, respectively) in comparison to WT. In contrast, the Suwalki variant showed a complex profile of variations, with overall fewer deviations than WT (Figure 9A). These results agree with those previously observed by CD, where the Merlo and Utrecht variants were the ones that lost the most secondary structure, while the Suwalki variant was the one with the highest content of secondary structure. However, the three variants with mutations affecting the Gly410 position showed slightly increased deviations compared with WT when averaged from three MDS replicas for each variant, suggesting no more remarkable changes in the overall folding. A higher RMSD value, as obtained for the G6PD Pro409 variants, indicates lower structural stability compared with WT-G6PD throughout the simulation. This correlates with experimental data, where catalytic activity was <1% for the Utrecht, Merlo, and Suwalki variants, possibly due to their structural instability. In contrast, kinetic parameters were obtained for the Gly410 variants.

Nevertheless, sidechain fluctuation analysis showed that several changes arose; for the Pro409 variants, the average RMSF values were similar for Merlo (av. 2.0 Å) and Utrecht (av. 1.9 Å), whereas Suwalki remained slightly higher, compared with WT (av. RMSF 2.5 vs. 1.8 Å). The increase in the RMSF value of the Pro409 variants was greater than 0.05 nm (0.5 Å) relative to WT-G6PD, indicating a substantial increase in amino acid flexibility [47], likely leading to loss of enzymatic structure and function. Moreover, Riverside, Shinagawa, and Kawasaki exhibited increased RMSF values around positions 200–220, compared with WT (Figure 9B), suggesting that sidechain reorganization plays a central role in turnover control (Figure S2). Important amino acids for G6PD enzyme catalysis are found at positions 200–220. The G6P substrate binding site comprises residues at positions 170–273, and the amino acids RIDHYLGKE (198–206 of human G6PD) are a conserved region in the G6PD enzyme of various species, whose sequence is important for catalysis [48]. Furthermore, Asp200, Hys201, and Lys205 have been shown to be important in G6P binding and catalysis in *L. mesenteroides*, and Lys205 has also been implicated in the binding and catalysis of the human enzyme. Overall, the MDS results show that mutations at amino acids 409 and 410 lead to structural alterations, decreased stability, and loss of catalytic efficiency, as obtained experimentally with the Kawasaki, Riverside, and Shinagawa variants, whose *k*_cat_ values decreased between 14 and 6 times relative to WT-G6PD.

**Figure 10:**
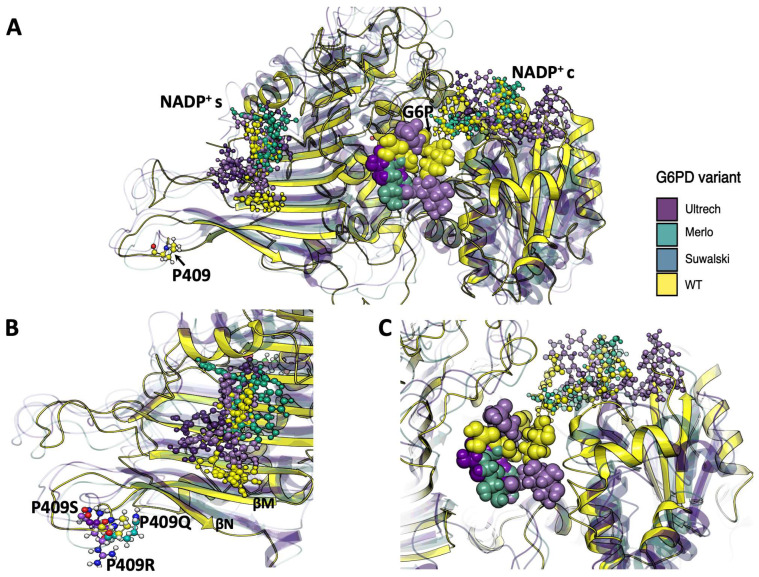
Overall superposition of MDS average structures of G6PD and Pro409 β-sheet variants with bound cofactors. (**A**) Overall schematic of G6PD variants bound to NADP^+^c and NADP^+^_s_. (**B**) Schematic of Pro409 variants in the NADP^+^_s_ region. (**C**) Schematic of Pro409 variants in the NADP^+^c region. NADP^+^ molecules are depicted as cpk spheres; G6P molecules are depicted as VDW spheres.

To gain insights into conformational changes derived from point mutations, the overall structure elements were compared with the WT system (Figure 10A). Monomer A of each variant was superimposed to visualize the changes in NADP^+^ location during the simulation, providing insights into the possible experimental loss of function on the heterologously expressed variants. For the Pro409 variants, the NADP^+^_s_ coordination sphere changed as the cofactor was displaced ca. 5 Å. The Suwalki and Utrecht variants displayed the most drastic rearrangements in the β-sheet loops (βN- and βM-sheets), with NADP^+^_s_ displacements of 4 and 5 Å, respectively, and a concomitant loss of NADP^+^_s_ coordination (Figure 10B). However, the catalytic NADP^+^c remained coordinated to Ser40, Asp42, Leu43, Arg72, S73, Tyr112, and Tyr249 and surrounded by several water molecules. Nonetheless, few G6P molecules were observed during the simulation time, suggesting a reduction in substrate binding affinity. Furthermore, the few G6P molecules that resided in the binding site could not dock in a catalytic orientation (Figure 10C). These results indicate that mutations at position 409 induce drastic conformational changes in the G6PD protein and, consequently, loss of function. This is consistent with the steady-state kinetic parameters, where <1% residual enzyme activity was detected due to a reduction in the substrate binding affinities and alterations of the secondary structure for the Utrecht, Merlo, and Suwalki variants.

**Figure 11:**
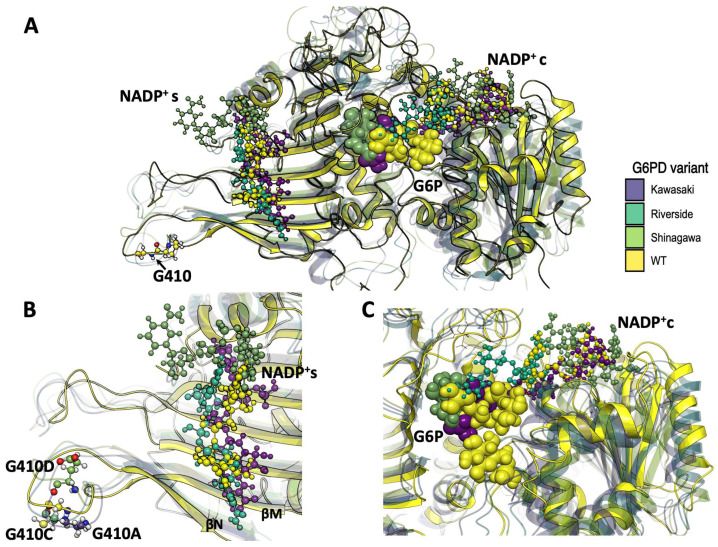
Overall superposition of MDS average structures of G6PD Gly410 β-sheet variants with bound cofactors. (**A**) Overall schematic of G6PD variants bound to NADP^+^c and NADP^+^_s_. (**B**) Schematic of Gly410 variants in the NADP^+^_s_ region. (**C**) Schematic of Gly410 variants in the NADP^+^c region. NADP^+^ molecules are depicted as cpk spheres; G6P molecules are depicted as VDW spheres.

For the Gly410 variants, the NADP^+^_s_ coordination sphere was also altered, but to a lesser extent, displaying a maximum cofactor displacement ca. 7 Å. (Figure 11A). The Kawasaki and Shinagawa variants displayed lower rearrangements in the β-sheet loops (βN- and βM-sheets), with an NADP^+^_s_ displacement of 2 and 3 Å, respectively, but without loss of NADP^+^_s_ coordination (Figure 11B). However, the NADP^+^c retained some elements of its coordination sphere, such as Leu43, Lys47, Arg72, and Tyr112, whereas Asp42 and Tyr249 were lost. Moreover, among the simulation replicates, very few G6P molecules were found in the active sites of the Kawasaki and Shinagawa variants. Interestingly, the Riverside variant showed the most dramatic changes in the NADP^+^_s_ β-sheet region, with 7 Å of displacement and almost a complete deformation of the cofactor (Figure 11B). This structural rearrangement resulted in no binding events during the cumulative 1.5 µs of simulation. It is worth mentioning that this variant showed a slight increase in stereogenic alpha carbon (C_α_) deviation and an NADP^+^c that was drastically displaced from its coordination sphere and filled with water molecules (Figure S3).

**Figure 12:**
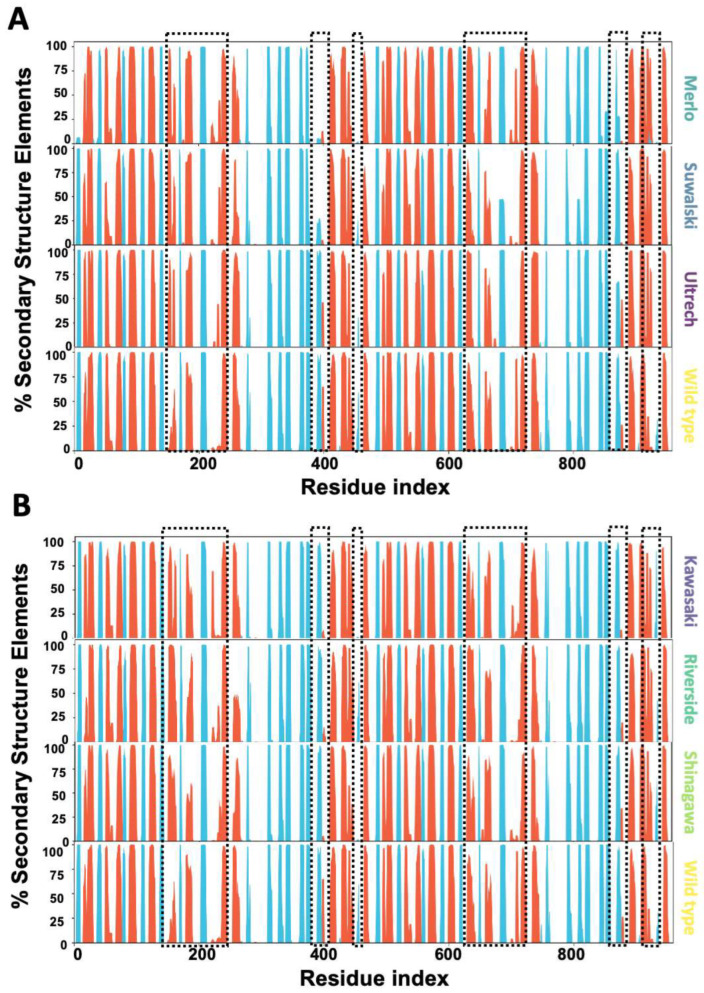
Percentage of the secondary structure elements of the whole sequence of G6PD β-sheet variants. (**A**) Pro409 variants’ %SSE. (**B**) Gly410 variants’ %SSE. ⍺-helical structures are depicted in red, and the β-sheets are depicted in cyan. Dotted boxes showed SEE with significant changes (<10%).

To further understand the conformational changes induced by point mutations affecting Pro409 and Gly410, we analyzed the secondary structure elements (%SSE) of the G6PD enzyme across the entire sequence. These changes were visualized as histograms reflecting the cumulative SSE profiles. The data revealed that local changes in β-sheet positions propagate changes in the binding site of G6P (195–205) and around the β-hairpin domain that contains the mutations (380–410). In the Merlo, Suwalki, and Utrecht variants, the percentage of α-helical structure in the 185–200 region increased significantly (70–95%) compared with WT (25–50%), while the β-sheet content around residue 200 remained unchanged (Figure 12A). Furthermore, the secondary structure elements around residue 400 also changed from 50% ⍺-helix and 95% β-sheet to less than 25% in Merlo and Suwalki variants.

**Figure 13:**
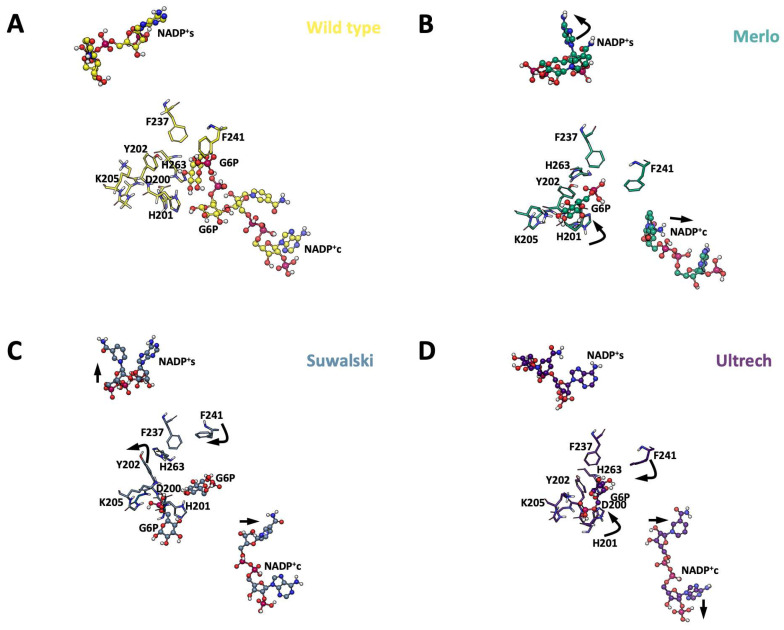
Structural perturbations displayed in Pro409 β-loop variants. (**A**) WT-G6PD. (**B**) G6PD Merlo. (**C**) G6PD Suwalki. (**D**) G6PD Utrecht. NADP^+^ is depicted as cpk spheres, and key G6P-binding amino acids are shown as sticks. Point mutations affecting the Pro409 codon promote rearrangements in the hydrogen bonds and electrostatic and hydrophobic interaction patterns around NADP^+^_c_. The black arrows indicate the direction of the main vector of motion of selected residues, including NADP^+^.

For the Gly410 variants, the data analysis showed that mutations in the β-sheet also modified the %SSE, with increases in the ⍺-helical region between 185 and 200 residues. Moreover, these variants also showed changes in the SSE around residue 400 when compared with WT (Figure 13B). These alterations around residue 400 probably are due to the amino acids in this region corresponding to the subunit interface in the enzymatically active G6PD dimer (aas 380–410), which might destabilize the 3D structure of the protein, increasing its thermolability and decreasing its catalytic activity. Our results suggest that mutations in the β-sheet impose allosteric control of G6P transformation, despite being located farther away than 20 Å (Figure S4).

Allosteric regulation of G6PD has been well documented for Class I and II mutations [44,45,46,49,50]. Wei et al. [46] report that G6PD activity is intricately regulated by a structural NADP^+^_s_ cofactor distal from the catalytic NADP^+^c site. With the use of cryo-electron microscopy (cryo-EM) and crystallographic studies, they elucidated how NADP^+^_s_ stabilizes G6PD oligomerization and allosterically modulates substrate binding. Moreover, the same report addressed several key structural changes that determine a fine interrelation between structural and biochemical elements that govern G6PD allosteric regulation and its implications for understanding G6PD deficiency disorders.

Conformational rearrangements in the NADP^+^_s_ binding domain propagate long-range structural changes that modify the catalytic site. We evaluated changes in region 201–205, which adopts an ⍺-helical conformation (f’ helix) upon NADP^+^_s_ binding, positioning H201 and Y202 towards G6P substrate molecules. Moreover, F237 and F241 also change their configuration towards the G6P binding site, preventing steric clashes and promoting substrate binding. Hence, these four positions were evaluated as key structural changes in the G6P binding site for all variants to gain insights into the allosteric regulation.

Our analyses showed that in all the Pro409 variants, H201 moves away from G6P, whereas F241 moves apart from NADP^+^c, constraining the volume of the binding cavity and reducing the probability of substrate binding. These changes were also accompanied by displacement of NADP^+^c away from the binding cavity. Interestingly, Y202 moves away from the binding site in the Suwalki variant, increasing the cavity volume and promoting coupling of the substrate. Nonetheless, both NADP^+^ molecules were displaced from their coordination spheres in this variant (Figure 13).

**Figure 14:**
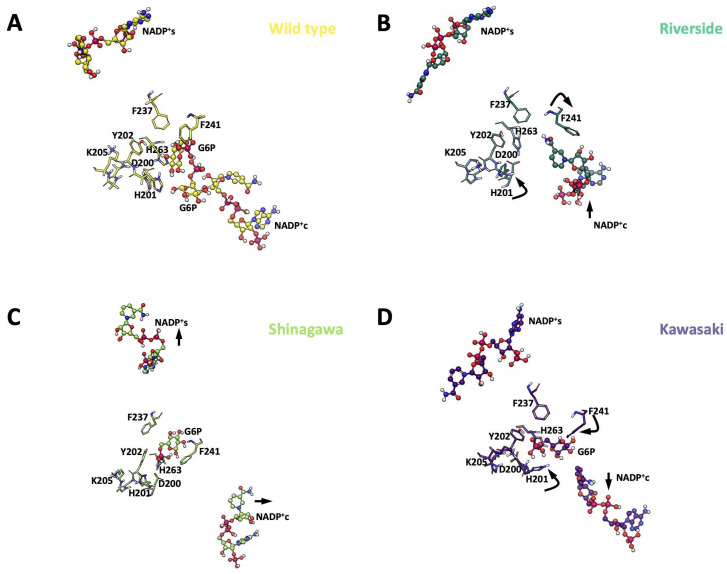
Structural perturbations displayed in Gly410 β-loop variants. (**A**) WT-G6PD. (**B**) G6PD Riverside. (**C**) G6PD Shingawa. (**D**) G6PD Kawasaki. NADP^+^ is depicted as cpk spheres, and key G6P-binding amino acids are shown as sticks. Point mutations affecting the Gly410 codon promote rearrangements in the hydrogen bonds and electrostatic and hydrophobic interaction patterns around NADP^+^_c_. The black arrows indicate the direction of the main vector of motion of selected residues, including NADP^+^.

The Gly410 variants displayed similar rearrangements in H201, where the azole side chain moved away from G6P, and F241 moved towards G6P. Again, these rearrangements were accompanied by displacement of NADP^+^c away from the binding cavity. In contrast to Pro409 variants, these mutants were able to accommodate G6P in the binding site, but in unproductive conformations (Figure 14).

The combination of kinetic analyses with structural insights derived from molecular dynamics simulations and spectroscopic methods enabled us to propose how specific amino acid substitutions at Pro409 and Gly410 modify the enzyme’s structure, resulting in distinct functional consequences. The complete loss of activity observed for the Pro409 variants highlights the critical role of this residue in G6PD function and stability. The molecular dynamics simulation analysis data provided deeper insight into the structural impact of the Pro409 mutations (Pro409Ser, Pro409Arg, Pro409Gln). Specifically, the substitutions at this position, which is located in a critical β-loop region close to the dimer interface and 20 Å from the active site, produced significant conformational rearrangements, increased flexibility, or even complete disrupted key secondary structural elements. Moreover, marked destabilization of the β-turn involving Pro409, which is crucial for maintaining the integrity of the substrate-binding pocket, concomitant with the introduction of a polar (Ser)/charged (Arg) residue at this hydrophobic/constrained position, led to severe steric clashes (Gln) and altered hydrogen-bonding networks.

In contrast, the Shinagawa variant (Gly410Asp) retained partial NADP^+^ coordination but showed loop rearrangement at the G6P active site, explaining its higher NADP^+^ affinity but reduced G6P affinity. Moreover, the Kawasaki (Gly410Ala) variant showed a marked effect, where the substitution of glycine with alanine, a slightly larger but non-polar residue, likely introduces minor steric hindrance or alters local packing. Intriguingly, G6PD Shinagawa (Gly410Asp) exhibited enhanced affinities for both G6P and NADP^+^. This unexpected functional outcome can be rationalized by considering the introduction of a negatively charged aspartate residue at position 410. Our MD simulations showed that the His201 azole side chain might form a transient interaction with a positively charged residue near the G6P or NADP^+^ binding pocket, which might reorient water molecules to an unproductive conformation. This could potentially destabilize the enzyme–substrate complex or increase the energetic barrier for substrate entry, thereby lowering the apparent *K*_m_ values. In contrast, G6PD Riverside (Gly410Cys) displayed significantly reduced affinities for both G6P and NADP^+^, suggesting severe impairment of substrate recognition and binding. The substitution of glycine with cysteine introduces a sulfhydryl group that is highly reactive and can participate in disulfide bond formation or alter local redox conditions. Moreover, the Riverside (Gly410Cys) variant simulations showed increased RMSF at residues 201–205 and changes in the hydrogen bond network within the catalytic NADP^+^ site, correlating with reduced affinity for both G6P and NADP^+^.

Furthermore, MD simulations provided crucial insights into this phenomenon, strengthening the hypothesis that conformational rearrangement of the NADP^+^ binding loop (residues 201–205) may cause disruption of critical interactions within the active site. Specifically, we observed (Figure 14) that the formation of a new hydrogen bond between the sulfhydryl group of Cys410 and Lys432 (Figure 4C) could pull the loop out of its optimal position or hinder the proper orientation of active site residues. This new interaction, or a more generalized disruption caused by the cysteine’s unique properties, likely perturbs the precise structural requirements for optimal substrate and co-factor binding, leading to the observed reduced affinities.

It is worth mentioning that the significantly altered thermostability profile of G6PD Riverside is consistent with a more conformationally unstable enzyme, which would also contribute to compromised substrate binding. Collectively, the distinct functional profiles of the Gly410 variants underscore the critical, yet nuanced, role of this residue in G6PD activity. Despite being at the same position, the nature of the amino acid substitution (small, non-polar, negatively charged, or thiol-containing) dictates the specific structural perturbations, which in turn manifest as unique kinetic outcomes.

Finally, structural rearrangements of distal portions of G6PD variants, as the region spanning residues 198–206 (RIDHYLGKE), a highly conserved peptide sequence identified as a crucial part of the catalytic NADP^+^ binding site, might be directly involved in substrate binding and catalysis within the N-terminal β-α-β domain of the G6PD enzyme [51]. Our data showed substantial impairment of activity and alteration of overall structure in all the studied variants in this region (more drastically in the Pro409 variants). For example, the Lys205 residue within this loop is critical for substrate binding and the catalytic mechanism. We found that, for this particular position, changes in distal residues (Gly410) can allosterically affect glucose-6-phosphate (G6P) binding and overall enzyme efficiency. Moreover, when the region encompassing residues 380–410, located within the C-terminal β+α domain, is disturbed, its pivotal role in the dimerization of G6PD monomer might be the main impairment, resulting in inactive monomers.

These findings highlight a sophisticated interplay between primary sequence changes and their impact on local protein dynamics, active site integrity, and substrate interactions, providing a molecular basis for the diverse clinical manifestations observed in patients with G6PD deficiency.

## 3. Materials and Methods

### 3.1. Phylogenetic Analysis of the Conservation of G6PD Protein Residues and Impact on G6PD Protein Structure

The phylogenetic conservation of G6PD protein residues was assessed using the ConSurf web server (https://consurf.tau.ac.il/consurf_index.php; accessed on 28 May 2024). This tool analyzes the phylogenetic relationships among diverse homologous G6PD sequences and calculates a conservation score ranging from 1 (most variable) to 9 (most conserved) [52,53]. To analyze the structural implications of the variants for the 3D structure of the G6PD protein, the HOPE server was used (https://www3.cmbi.umcn.nl/hope/; consulted on 28 May 2024). HOPE relies on several sources to acquire relevant data and predict the implications of mutation on the structure and function of the examined protein [24]. The analysis was performed using the G6PD protein structure from PDB ID: 2BH9.

#### In Silico Site-Directed Mutagenesis

Single amino acid mutation was performed on the G6PD enzyme structure to generate the G6PD Suwalki (Pro409Arg), Merlo (Pro409Gln), Utrecht (Pro409Ser), Kawasaki (Gly410Ala), Riverside (Gly410Cys), and Shinagawa (Gly410Asp) variants. Mutagenesis was performed using UCSF Chimera v1.16 [54] employing the Rotamers tool and the Dunbrack 2010 backbone-dependent rotamer library [55]. The residues Pro409 and Gly410 were substituted with the corresponding amino acid in each case, and the resulting mutant models were subjected to energy minimization using the locPREFMD algorithm [56]. The graphical representations of the resulting mutant models were prepared with UCSF Chimera v1.16, and the file was saved as a PDB.

### 3.2. Site-Directed Mutagenesis

To generate the desired mutations in the *G6PD* gene, the corresponding specific mutagenic oligonucleotides were designed from the Gen Bank sequence (NM_001042351) to create the six clinical natural mutants: G6PD Suwalki (Pro409Arg), Merlo (Pro409Gln), Utrecht (Pro409Ser), Kawasaki (Gly410Ala), Riverside (Gly410Cys), and Shinagawa (Gly410Asp). Table 2 shows the mutagenic oligonucleotides, which consist of 20 base pairs (bp) that were chemically synthesized at the Unidad de Síntesis y Secuenciación del Instituto de Biotecnología (IBT) of the UNAM.

To carry out the construction of the six clinical G6PD mutants, PCR site-directed mutagenesis was performed using the Quick-Change technique, with the pET-3a expression plasmid as a template, which contains the wild *G6PD* gene. PCR was performed as previously reported by Martínez-Rosas et al. [39]. The PCR products were digested with the *Dpn*I enzyme for 4 h at 37 °C, and the digestion product was transformed into competent *E. coli* TOP-10 cells. Subsequently, the plasmid was purified from each transformed colony with a GeneJET Plasmid Miniprep^®^ Kit (Thermo Fisher Scientific, Waltham, MA, USA). To corroborate the fidelity of each of the mutations and to confirm that they did not contain undesired mutations, the plasmids were sequenced at the Unidad de Síntesis y Secuenciación del Instituto de Biotecnología (IBT) of the UNAM. Once the presence of each mutation was confirmed, the plasmids were transformed into competent *E. coli* BL21(DE3)Δzwf::kan^r^ cells. Transformed colonies were selected on Luria–Bertani (LB) agar plates containing ampicillin (100 µg/mL) and kanamycin (100 µg/mL).

### 3.3. Purification of G6PD Variants

*E. coli* BL21(DE3)Δzwf::kan^r^ cells containing the plasmids with the desired mutations were grown in 2 L of LB liquid medium containing ampicillin (100 µg/mL) and kanamycin (100 µg/mL) with constant agitation at 37 °C. When the culture reached an optical density (OD600) of 0.8, protein expression was induced by adding 0.3 mM isopropyl β-D-1-thiogalactopyranoside (IPTG). Cultures were incubated under agitation for 18 h. Subsequently, the cells were centrifuged and lysed by sonication, as previously described by Gómez-Manzo et al. [1]. The lysate was centrifuged at 9000 rpm for 30 min, and the resulting supernatant (referred to as the crude extract) was collected and assayed for catalytic activity.

The standard reaction mixture contains 1 mM G6P and 1 mM NADP^+^ dissolved in buffer T (100 mM Tris-HCl, 3 mM MgCl_2_ at pH 8.0). The catalytic activity was measured at 340 nm and 25 °C spectrophotometrically, measuring the production of NADPH. Purification of recombinant G6PD enzymes was performed as previously described using 2′,5′-ADP Sepharose 4B affinity and anion exchange Q-Sepharose-4B columns [1,9,35,36,37,38,39,40]. The purity of WT-G6PD and the variants was analyzed on 12% SDS-PAGE gels stained with colloidal Coomassie Brilliant Blue (Sigma-Aldrich, St. Louis, MO, USA), while the protein concentration was determined by Lowry assay [57] using bovine serum albumin (BSA) as the standard. The recombinant G6PD proteins were used immediately after being purified.

### 3.4. Functional Characterization Assays

#### 3.4.1. Steady-State Kinetics

Steady-state kinetic parameters for WT-G6PD and the clinical variants (Suwalki, Merlo, Utrecht, Kawasaki, Riverside, and Shinagawa) were determined by monitoring NADPH production at 340 nm. Initial velocities were measured by varying the concentration of one substrate (G6P or NADP^+^) from 2.5 to 200 µM, while the second substrate (G6P or NADP^+^) was fixed at a saturating concentration (1 mM). For the WT-G6PD enzyme, the reaction was initiated by the addition of 200 ng of G6PD, while those for G6PD variants were initiated by adding 1 µg of purified protein. The reaction was initiated with the addition of 1 µg of pure protein of each variant. The initial velocities were used to calculate NADPH formation rates (µmol/min/mg) using the NADPH extinction coefficient at 340 nm (ε = 6220 M^−1^ cm^−1^). Kinetic parameters (K_m_, *k*_cat_, and *V*_max_) were obtained by non-linear regression fitting to the Michaelis–Menten equation [1,9,36,37,38,39].

#### 3.4.2. Thermal Inactivation Assay

A thermal inactivation assay was carried out with the purpose of evaluating the effect of mutations on the stability of WT-G6PD, using as a reference the loss of catalytic activity when they are incubated at different temperatures. The proteins were adjusted to a concentration of 0.2 mg/mL and were incubated in a temperature gradient from 37 to 61 °C for 20 min. Subsequently, the samples were cooled to 37 °C for 5 min, and the residual activity of each of the G6PD variants was immediately measured using a final volume of 1 mL of the reaction mixture (100 mM Tris-HCl, 3 mM MgCl_2_, 1 mM G6P, and 1 mM NADP^+^). Residual enzyme activity was measured in a spectrophotometer at 340 nm and expressed as a percentage of the residual enzyme activity. The T_50_ value (temperature at which 50% of the initial activity is lost) was calculated from these data.

### 3.5. Spectroscopic Characterization Assays

#### 3.5.1. Circular Dichroism

To evaluate secondary structural changes in the G6PD variants, circular dichroism (CD) spectroscopy was performed. To carry out the assays for each of the G6PD variants, each variant was dialyzed in a 25 mM phosphate-buffer solution, pH 7.35, and adjusted to a final protein concentration of 0.4 mg/mL. The CD analysis was performed in a spectropolarimeter (Jasco J-810^®^, Easton, MD, USA) equipped with a Peltier thermostatted cell holder. Far UV-CD spectra of the six clinical G6PD variants were detected from 200–260 nm at 1 nm intervals in a quartz cell with a path length of 0.1 cm, as previously described [1,9,36,37,38,39,40]. The blank (phosphate buffer) was subtracted from each of the spectra. All experiments were conducted in triplicate at 25 °C.

#### 3.5.2. Intrinsic and Extrinsic Fluorescence

Intrinsic and extrinsic fluorescence assays were conducted to evaluate the effects of mutations on the tertiary structure of the G6PD variants. For both assays, a Perkin-Elmer L-45^®^ fluorescence spectrometer (Perkin Elmer, Wellesley, MA, USA) was used, equipped with a quartz cell with a step length of 1 cm. For both intrinsic and extrinsic fluorescence assays, a protein concentration of 0.1 mg/mL was used for WT-G6PD and for each of the six clinical G6PD variants. The proteins were diluted in 25 mM K_2_HPO_4_ buffer at pH 7.35. For the intrinsic fluorescence assay, the samples were excited at 295 nm, and data on the fluorescence emission from 310 to 500 nm were collected. For the extrinsic fluorescence assay, the proteins were incubated with 1-aniline-8 naphthalene sulfonate at 165 µM. Subsequently, the samples were excited at 395 nm, and the emission data were collected within an emission range of 400 to 600 nm. Excitation and emission slits (apertures) of 5 and 5 nm were used, respectively. In both assays, the blank spectra were subtracted (25 mM K2HPO4 buffer at pH 7.35).

### 3.6. Molecular Dynamics Simulations (MDSs)

#### General Simulation Setup and Parameterization

MDSs were performed using a Maestro 2024-3 (New York, NY, USA; Version 13.8.135, MMshare Version 6.4.135, Release 2023-4) in combination with the Desmond Multisim v4.0.0 interoperability tools package—Academica version 5.6 (New York, NY, USA). The dimeric structure of G6PD in complex with NADP^+^ was obtained from the Protein Data Bank (PDB ID: 2BH9) [51]. Single point mutations affecting residues Pro409 and Gly410 were introduced using Maestro v2024-3. Protein, ion, and ligand parameters were assigned using the OPLS-3 force field [58]. The atomic charge distribution for the NADP^+^ cofactors was refined based on previously described ab initio calculations and fitted to RESP charges [59]. All systems were solvated with TIP3P water molecules and neutralized with 0.15 M NaCl or KCl. The final systems contained approximately 435,000 atoms in a cubic box of 60 × 60 × 75 Å.

Initially, the systems were minimized to 50,000 steps calculated with the steepest-descendent method, followed by 25,000 steps with the conjugate gradient method, until they reached energy minima. Subsequent gradual heating simulations were performed under the NVT ensemble coupled to a Nosé–Hoover thermostat [60] at 310 K, with linear temperature ramps from 0 to 310 K for 15 ns. To achieve structural equilibration, further MD simulations using the isothermal-isobaric ensemble (NPT) were performed by applying a Martyna–Tobias–Klein barostat [61,62] to maintain a pressure of 1 atm at 310 K for 50 ns. Long-range electrostatic interactions were calculated under periodic boundary conditions (PBC) using the particle mesh Ewald (PME) method [63], with a cut-off distance of 12 Å and a force switch at 9 Å. Three independent simulations of each variant system were performed for 0.5 µs each (1.5 µs cumulatively), with integration time steps set to 2.0 fs. For each replica, the root mean square deviation (RMSD) of the stereogenic amide center (alpha carbons, C⍺) and the root mean square fluctuations of side chains (beta carbons, Cβ) were calculated with Schrödinger *Maestro* v 2024-3 simulation integration diagram tools for at least five residues from the N-terminus and C-terminus and averaged to evaluate convergence of the simulations. To gain detailed insights into the structural and functional consequences of the identified G6PD mutations, our molecular dynamics simulations and subsequent analyses focused on specific regions known to be critical for enzyme activity and stability. These regions include residues 201–205 and 380–410; however, the secondary structure elements were extracted from full trajectories and expressed as percentages.

## 4. Conclusions

This study provides an integrated biochemical and computational characterization of six clinical G6PD variants located at Pro409 and Gly410, two residues positioned within the β-loop near the structural NADP^+^ (NADP^+^_s_) site. Our findings demonstrate that these mutations severely compromise G6PD function through distinct yet converging mechanisms. Variants affecting Pro409 (Suwalki, Merlo, and Utrecht) exhibited enzyme activity < 1%, reduced thermal stability, and profound secondary and tertiary structural alterations. These effects are attributable to disruption of the β-turn motif essential for oligomer folding and NADP^+^_s_ coordination. In contrast, variants affecting Gly410 (Kawasaki, Riverside, and Shinagawa) retained partial enzymatic activity but exhibited compromised substrate binding, reduced stability, and NADP^+^_s_ displacement. Molecular dynamics simulations further revealed that these point mutations propagate allosteric changes from the NADP^+^_s_ site to the catalytic core, affecting cofactor orientation, substrate accessibility, and secondary structure elements critical for function. The coupling between conformational stability and catalytic efficiency underscores the importance of the NADP^+^_s_ region as a regulatory motif. Altogether, our findings offer a mechanistic basis for the severe phenotypes observed in patients with these variants and reinforce the structural NADP^+^ site as a strategic target for future pharmacological stabilization or gene therapy approaches for G6PD deficiency.

## Figures and Tables

**Table 1:** Summary of the kinetic parameters of WT-G6PD and six clinical G6PD mutants.

G6PD	Class	Amino Acid Substitution	*k*_cat_ (s^−1^)	K_m G6P_ (µM)	K_m NADP_^+^ (µM)	*k*_cat_/K_m G6P_ (µM^−1^ s^−1^)	*k*_cat_/K_m NADP_^+^ (µM^−1^ s^−1^)
WT	-		211.3 ± 3.1	42.8 ± 1.7	7.8 ± 0.3	4.9 ± 0.3	27.1 ± 1.9
Suwalki	A	Pro409Arg	ND	ND	ND	ND	ND
Merlo	A	Pro409Gln	ND	ND	ND	ND	ND
Utrecht	A	Pro409Ser	ND	ND	ND	ND	ND
Kawasaki	I	Gly410Ala	34.3 ± 1.2	32 ± 1.3	9.1 ± 1.3	1.1 ± 0.05	3.8 ± 0.2
Riverside	I	Gly410Cys	18.6 ± 1.8	176.8 ± 8.2	15.6 ± 0.7	0.10 ± 0.005	1.19 ± 0.05
Shinagawa	I	Gly410Asp	18.7 ± 0.3	11.8 ± 0.5	3.7 ± 0.2	1.5 ± 0.06	5.1 ± 0.18

ND—G6PD activity < 1%.

**Table 2:** Mutagenic oligonucleotides to obtain the mutants used in this study.

Mutant	Mutagenic Oligonucleotide	Reference
Utrecht	Fw: 5′ CCAAGAAGTCGGGCATGTTC-3′ Rv: 5′ GGTTCTTCAGCCCGTACAAG-3′	This study
Suwalki	Fw: 5′ CCAAGAAGCGGGGCATGTTC-3′ Rv: 5′ GGTTCTTCGCCCCGTACAAG-3′	This study
Merlo	Fw: 5′ CCAAGAAGCAGGGCATGTTC-3′ Rv: 5′ GGTTCTTCGTCCCGTACAAG-3′	This study
Riverside	Fw: 5′-CCAAGAAGCCGTGCATGTTC-3′ Rv:5′-GAACATGCACGGCTTCTTGG-3′	This study
Kawasaki	Fw: 5′-CCAAGAAGCCGGCCATGTTC-3′ Rv: 5′-GAACATGGCCGGCTTCTTGG-3′	This study
Shinagawa	Fw: 5′-CCAAGAAGCCGGACATGTTC-3′ Rv: 5′-GAACATGTCCGGCTTCTTGG-3′	This study

## Data Availability

Data are contained within the article.

## Supplementary Materials

The following supporting information can be downloaded from https://www.mdpi.com/article/10.3390/ijms26178464/s1.
