# Supplementary material for: Integrated Approach for Biochemical and Functional Characterization of Six Clinical Variants of Glucose-6-Phosphate Dehydrogenase
Source: Int J Mol Sci. 2025 Aug 30;26(17):8464. doi: 10.3390/ijms26178464 (PMC12429737; doi:10.3390/ijms26178464)
Supplement: Supplementary file 1 [file ijms-26-08464-s001.zip › ijms-3791008-supplementary.pdf]

Table S1. Purfication summary of the recombinant human Wild-type (WT-G6PD) and the six variant enzymes.

G6PD	Total Protein (mg)	Specific Activity (IU·mg–1)	Total Activity (IU)	Yield (%)
WT	4.9	153	749	60
Suwalki	0.2	ND	ND	ND
Merlo	0.64	ND	ND	ND
Utrecht	0.1	ND	ND	ND
Kawasaki	4.4	44	193	23
Riverside	0.64	8	5.1	3
Shinagawa	1.28	14	1.28	4

ND = non-detected.

Figure S1

Figure S1. DDMUT analysis on G6PD P409 and G410 codons.

Codon P409

Results

Predicted Stability Change ($\Delta\Delta G^{Stability}_{wt\text{->}mt}$)

0.08 kcal/mol

(Stabilising)

Mutation Details

Chain: **A**
Position: **409**
Wild-type: **P**
Mutant: **S**

Wild-type residue environment

Depth: **1.92 Å**
RSA: **0.63 Å^3**
Phi: **-55.8 °**
Psi: **139.1 °**
Relative bfactor: **3.38**

Results

Predicted Stability Change ($\Delta\Delta G^{Stability}_{wt\text{->}mt}$)

0.1 kcal/mol

(Stabilising)

Reverse Prediction ($\Delta\Delta G^{Stability\ mt\text{->}wt}$)

-0.02 kcal/mol (Destabilising)

Mutation Details

Chain: **A**
Position: **409**
Wild-type: **P**
Mutant: **R**

Wild-type residue environment

Depth: **1.92 Å**
RSA: **0.63 Å^3**
Phi: **-55.8 °**
Psi: **139.1 °**
Relative bfactor: **3.38**

Results

Predicted Stability Change ($\Delta\Delta G^{Stability}_{wt\text{->}mt}$)

0.08 kcal/mol

(Stabilising)

Reverse Prediction ($\Delta\Delta G^{Stability\ mt\text{->}wt}$)

0.08 kcal/mol (Stabilising)

Mutation Details

Chain: **A**
Position: **409**
Wild-type: **P**
Mutant: **Q**

Wild-type residue environment

Depth: **1.92 Å**
RSA: **0.63 Å^3**
Phi: **-55.8 °**
Psi: **139.1 °**
Relative bfactor: **3.38**

Codon G410

Results

Predicted Stability Change ($\Delta\Delta G^{Stability}_{wt\text{->}mt}$)

0.06 kcal/mol

(Stabilising)

Reverse Prediction ($\Delta\Delta G^{Stability\ mt\text{->}wt}$)

0.07 kcal/mol (Stabilising)

Mutation Details

Chain: **A**
Position: **410**
Wild-type: **G**
Mutant: **D**

Wild-type residue environment

Depth: **1.71 Å**
RSA: **0.68 Å^3**
Phi: **102.3 °**
Psi: **-152.0 °**
Relative bfactor: **3.39**

Results

Predicted Stability Change ($\Delta\Delta G^{Stability}_{wt\text{->}mt}$)

-0.01 kcal/mol

(Destabilising)

Reverse Prediction ($\Delta\Delta G^{Stability\ mt\text{->}wt}$)

0.01 kcal/mol (Stabilising)

Mutation Details

Chain: **A**
Position: **410**
Wild-type: **G**
Mutant: **C**

Wild-type residue environment

Depth: **1.71 Å**
RSA: **0.68 Å^3**
Phi: **102.3 °**
Psi: **-152.0 °**
Relative bfactor: **3.39**

Results

Predicted Stability Change ($\Delta\Delta G^{Stability}_{wt\text{->}mt}$)

-0.11 kcal/mol

(Destabilising)

Mutation Details

Chain: **A**
Position: **410**
Wild-type: **G**
Mutant: **A**

Wild-type residue environment

Depth: **1.71 Å**
RSA: **0.68 Å^3**
Phi: **102.3 °**
Psi: **-152.0 °**
Relative bfactor: **3.39**

Figure S2.

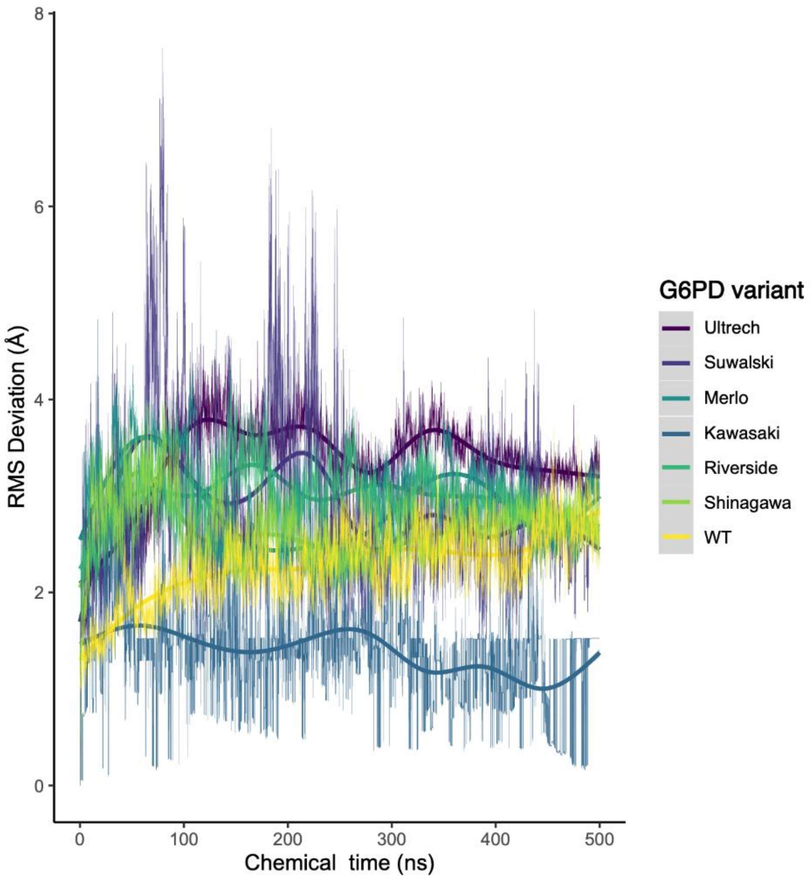

8
6
4
2
0
RMS Deviation (Å)
0
100
200
300
400
500
Chemical time (ns)
G6PD variant
Ultrech
Suwalski
Merlo
Kawasaki
Riverside
Shinagawa
WT

Figure S3

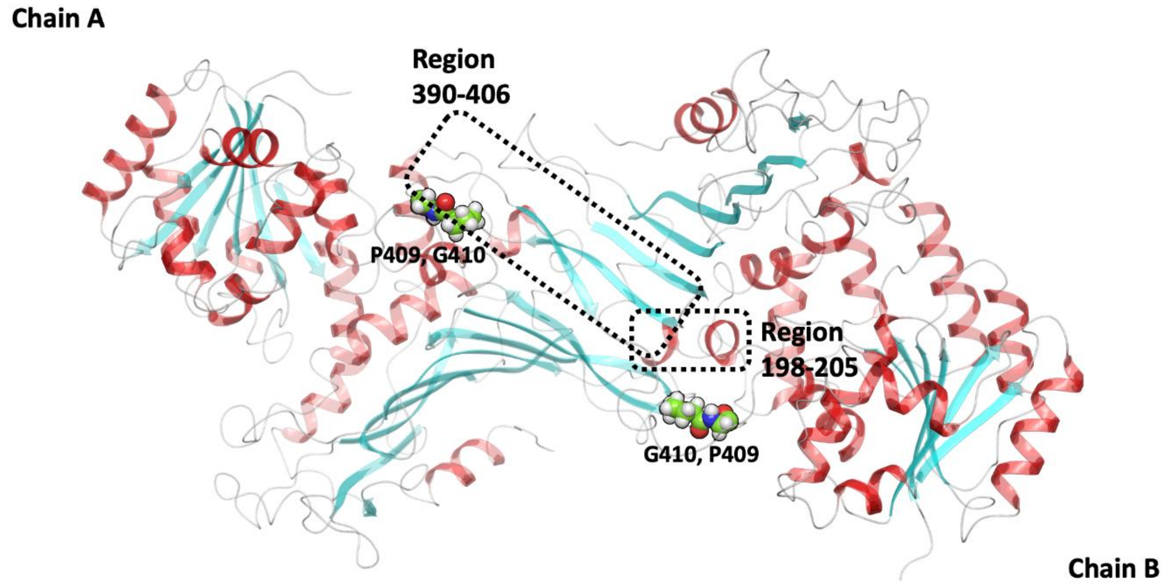
Chain A
Region
390-406
P409, G410
Region
198-205
G410, P409
Chain B

Figure S4.

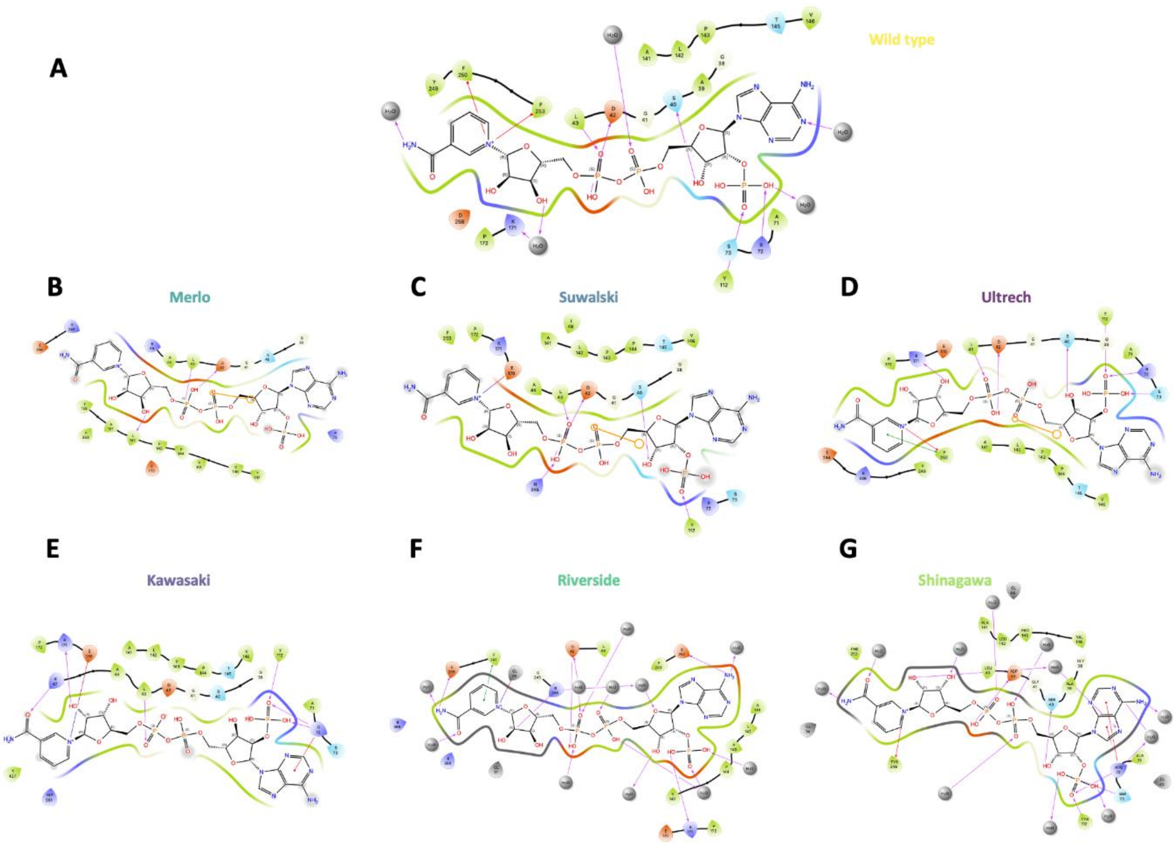
A
Wild type
B
Merlo
C
Suwalski
D
Ultrech
E
Kawasaki
F
Riverside
G
Shinagawa
